# Psychological side effects of hormonal contraception: a disconnect between patients and providers

**DOI:** 10.1186/s40834-022-00204-w

**Published:** 2023-01-17

**Authors:** Sarah Martell, Christina Marini, Cathy A. Kondas, Allison B. Deutch

**Affiliations:** 1 NYU Grossman School of Medicine, New York, NY USA; 2 Department of Psychiatry, Zucker Hillside Hospital, Queens, NY USA; 3 Department of Psychiatry, NYU Grossman School of Medicine, New York, NY USA; 4 Division of Consultation-Liaison Psychiatry, Department of Psychiatry, NYC Health + Hospitals | Bellevue, New York, NY USA; 5 Division of Consultation-Liaison Psychiatry, Department of Psychiatry, NYU Grossman School of Medicine, NY New York, USA

**Keywords:** Hormonal contraception, Birth control, Side effects, Contraception counseling, Mood changes

## Abstract

**Background:**

Existing literature about the psychological side effects of hormonal contraception (HC) is limited. The goal of this study is to better characterize patients’ subjective experiences with HC, its side effects, and contraception counseling.

**Methods:**

This is a cross-sectional, survey-based study using a convenience sample of patients who had used HC at some point in their lives. Recruitment occurred from June 2021-February 2022.

**Results:**

Of the 188 responses included in the analysis, 43.6% reported experiencing mood changes as a side effect of HC at some point in their lives. The most common reason participants cited for discontinuing or switching contraception methods was side effects (48.3%). Participants with a history of psychiatric illness were significantly more likely to report mood changes as a side effect of their HC (61.2%) compared to participants with no history of psychiatric illness (29.5%). Among patients with a history of psychiatric illness, 38.8% responded that their psychiatric symptoms worsened with HC while only 11.2% responded that their symptoms improved with HC. The majority (83%) of participants responded that their provider never mentioned the possibility of psychological side effects during contraception counseling. If/when they experienced side effects associated with their HC, 22.7% of participants disagreed that their provider adequately addressed their concerns.

**Conclusion:**

These findings suggest that mood changes may be among the most common perceived side effects of HC and speak to a disconnect between patients and providers when it comes to discussing the possibility of psychological side effects with HC.

**Supplementary Information:**

The online version contains supplementary material available at 10.1186/s40834-022-00204-w.

## Background

### Hormonal contraception and its side effects

Approximately 250 million people worldwide use some form of systemic hormonal contraception (HC). A 2013 report found that side effects were the most common reason women stopped using HC methods [1]. Other studies have suggested that psychological and sexual side effects are among the leading reasons for non-adherence or discontinuation of HC [2, 3].

### Hormonal contraception in the media

There is growing concern that media portrayals of hormonal birth control (BC) induced mood changes and depression may make women less likely to use birth control even in situations where it might be clinically indicated. Previous research investigating the role of the media in women’s contraceptive choices, has suggested that media played an important role in previous “Pill Scares,” in which negative media coverage surrounding HC and its side effects has resulted in women around the world discontinuing their HC [4]. The National Curriculum for Reproductive Psychiatry specifically references portrayals of HC in the popular media in their published online curriculum [5]. In one such media article the author suggests that physicians who downplay the burden of psychological side effects with HC are participating in the “medical gaslighting” of women. Facilitators of the curriculum are specifically asked to help trainees evaluate the ways in which media portrayals may negatively influence women’s perception of the safety and suitability of HC.

Some physicians and mental health providers have been sounding the alarm about psychological side effects of HC in books and TED talks, advocating for women to switch to non-hormonal methods of BC including the fertility awareness method (also known as “The Rhythm”) [6–8]. These conversations are associated with a movement away from HC towards non-hormonal, less effective methods of BC [9]. For example, fertility awareness apps such as *Natural Cycles*, marketed for pregnancy prevention, have been gaining popularity - with over 2 million registered users.

### Existing literature about psychological side effects of hormonal contraception

In 1974, a longitudinal study of nearly 46,000 women, conducted by Kay et al. reported a 30% increase in depressive symptoms and decreased libido in women taking oral contraceptive pills (OCPs). Psychological symptoms improved after discontinuing OCPs in this sample [10]. More recently, Skovlund and colleagues conducted a longitudinal prospective study of one million women in Denmark and found that use of HC was associated with increased risk of first diagnosis of depression and subsequent treatment with antidepressants [11]. A follow-up study focused on adolescents included half a million women who turned 15 during the study period and had no prior psychiatric diagnoses. They found that HC use was associated with a two-fold increased relative risk (RR) of suicide attempts and a three-fold increased RR of completed suicide [12].

Despite this data, the relationship between HC and psychological side effects remains controversial. Recent review articles on the subject have identified several limitations in the existing literature including a relative lack of prospective studies, a variety of methods to define mood changes, and failure to differentiate patients by the type of HC used [13]. Several reviews have concluded there is a low incidence of adverse mood changes with HC in the general population and that the effects of HC on mood appear to be most relevant in selected subsets of women including those with a history of depression, previous negative experiences with HC, and adolescents [13–16].

### Guidelines for counseling and study aims

Anticipatory guidance and shared decision making with informed consent are critical for developing and maintaining a therapeutic alliance and improving adherence. However, there are mixed messages in the media and academic literature about the burden of psychological side effects of HC. Existing guidelines for contraception counseling also fail to address the subject of psychological side effects, offering providers little guidance on how to counsel patients with these concerns [17–20].

To better understand patients’ experience(s) with HC and contraception counseling, we conducted a cross-sectional survey-based study to characterize patients’ experiences with medical providers as it relates to the use of HC, perceived side effects, and reasons for discontinuation.

## Methods

### Study design

A cross-sectional study was conducted anonymously via an online RedCap survey. Recruitment occurred from June 2021 through February 2022.

### Setting

The survey was administered online in English-language only. The survey took approximately 10 min to complete.

### Participants

Adults (age > 18) who had ever used HC were eligible to participate in the study. The survey was voluntary and anonymous. Participants did not receive compensation for participation. The survey was distributed via social media, email, word of mouth, and through flyers hung in women’s health clinics in New York City. The flyers contained a QR code so that subjects could access the survey on their smartphones. Investigators also asked their contacts to forward the survey link and post it in community forums such as sorority Facebook groups.

### Data sources/survey instrument

The survey was designed by study investigators to limit the amount of personal or identifying information collected. Investigators also tried to keep the survey brief (< 15 min) to maximize survey completion. All questions in the survey were optional and participants could stop taking the survey at any time. The majority of the survey questions were multiple choice, some utilized a likert scale, and some were free-response. Regarding psychiatric history, participants were asked if they had ever been diagnosed with or treated for a psychiatric illness. If they answered yes, they were asked to specify in a free response question. Responses were coded and analyzed quantitatively.

In order to keep the survey brief and to avoid asking participants for a timeline of their lifelong contraceptive choices, the survey asks participants only about the first method of BC they used, the one they are currently using, and which methods they have used throughout their lifetime. The survey does not distinguish between hormonal intra-uterine devices (IUDs) and copper IUDs. The complete survey instrument is included in Supplemental materials.

### Data analysis

Surveys with > 50% missing data were excluded from analysis. Data was exported from RedCap and all data cleaning and analysis was conducted using Python (Python Software Foundation v. 3.9). Descriptive statistics were used. Qualitative data was edited for typos and coded for themes. Two team members separately reviewed the qualitative data and verified the coding for data quality assurance. Two-sample t-tests were used to compare rates of reported side effects between participants with a history of psychiatric illness and those without a psychiatric history. If participants responded that they had ever been diagnosed or treated for a psychiatric condition, they were given the option to specify their diagnoses in a free response question.

### Ethical review

All study materials and methods were reviewed and deemed exempt from ongoing institutional review board (IRB) review by the New York University and Bellevue Hospital IRBs (i21-00663). A consent form was included at the start of the survey and consent was implied by participation in the study.

## Results

### Demographics

A total of 215 participants completed the consent form. Of these, 25 participants completed less than half of the survey, and 2 participants had never used HC. These 27 responses were excluded, leaving 188 responses included in the final analysis.

Table 1 displays the demographics and background information about respondents. Mean age was 31.9 years (Standard Deviation (SD): 9.1, Range: 22–72). A majority of respondents were white (86%, 160/186), and many were graduate students (26.1%, *n* = 49) and/or healthcare workers (27.1%, *n* = 51). The majority of participants (52.7%, 99/188) were unmarried and sexually active. 26% (49/188) of participants responded that they have been pregnant at some point. Of the participants who have been pregnant, the mean parity was 1.4 (SD: 1.1, Range: 0–5).

**Table 1:** Demographics of participants

	**n**	**%**
**Elective Abortions (*N* = 48)**	10	20.8
**Gender (*N* = 188)**		
Cisgender Female	183	97.3
Non-binary	5	2.7
**Race/Ethnicity (*N* = 186)**		
White	160	86.0
Asian	22	11.8
Hispanic/Latinx	14	7.5
Black	8	4.3
Middle Eastern	4	2.2
Other	3	1.6
**Sexuality (*N* = 187)**		
Heterosexual	141	75.4
Bisexual	35	18.7
Queer	6	3.2
Homosexual	4	2.1
Asexual	1	0.5
**Occupation (*N* = 188)**		
Employed	132	70.2
Student	50	26.6
Unemployed/Retired	14	7.4
**Marital Status (*N* = 188)**		
Single, sexually active	99	52.7
Married	67	35.6
Single, not sexually active	19	10.1
Divorced	2	1.1
Other	1	0.5

### Contraception methods and side effects

Table 2 displays the types of contraception used by study participants. The mean age of starting HC was 18.4 years (SD: 3.2, Range: 11–37). A majority (87.7%, 164/187) used OCPs as their first form of HC. The most common first prescriber of HC was gynecologists (70%, 128/188). Most participants started using HC for pregnancy prevention (71.8%, 135/188). Other common reasons for starting HC included dysmenorrhea (26.1%, 49/188) and menorrhagia (21.8%, 41/188). 

The most common reason participants cited for discontinuing or switching methods of contraception was side effects (48.3%, 70/145). Participants who switched methods due to concerns about hormones cited reasons including “desire to become more in tune with my body” and “belief from cultural messaging that [HC] wasn’t ‘natural’”.

**Table 2:** Contraception Data – Information about participants’ initial contraceptive method and responses to the question “If you have switched contraception methods or stopped using hormonal contraception, what was your reason?”

	**n**	**%**
***First HC Used (N = 187)***		
OCPs	164	87.7
IUD	12	6.4
Depoprovera	4	2.1
Nuvaring	4	2.1
Nexplenon	3	1.6
The Patch	2	1.1
**First Prescriber of HC (*N *= 188)**		
Gynecologist	124	70.0
Primary Care Provider	42	22.3
Pediatrician	14	7.4
Other	8	5.6
**Reason for Initiating HC (*N *= 188)**		
Pregnancy Prevention	135	71.8
Painful Periods	49	26.1
Heavy Periods	41	21.8
Acne	34	18.1
Irregular Periods	32	17.0
Premenstrual Symptoms	17	9.0
Ovarian Cysts	4	2.1
PCOS	2	1.1
**Reasons for Discontinuing/Switching Methods (*N *= 145)**		
Side Effects	70	48.3
Ease of Use	40	27.6
Desire to become pregnant	34	23.4
No longer at risk of pregnancy	19	13.1
Loss of access	10	6.9
Menopause	4	2.8
Concerns about hormones	4	2.8
Switched to more effective/long-term option	4	2.8
Migraines	4	2.8
Cost	1	0.7

**Fig. 1:**
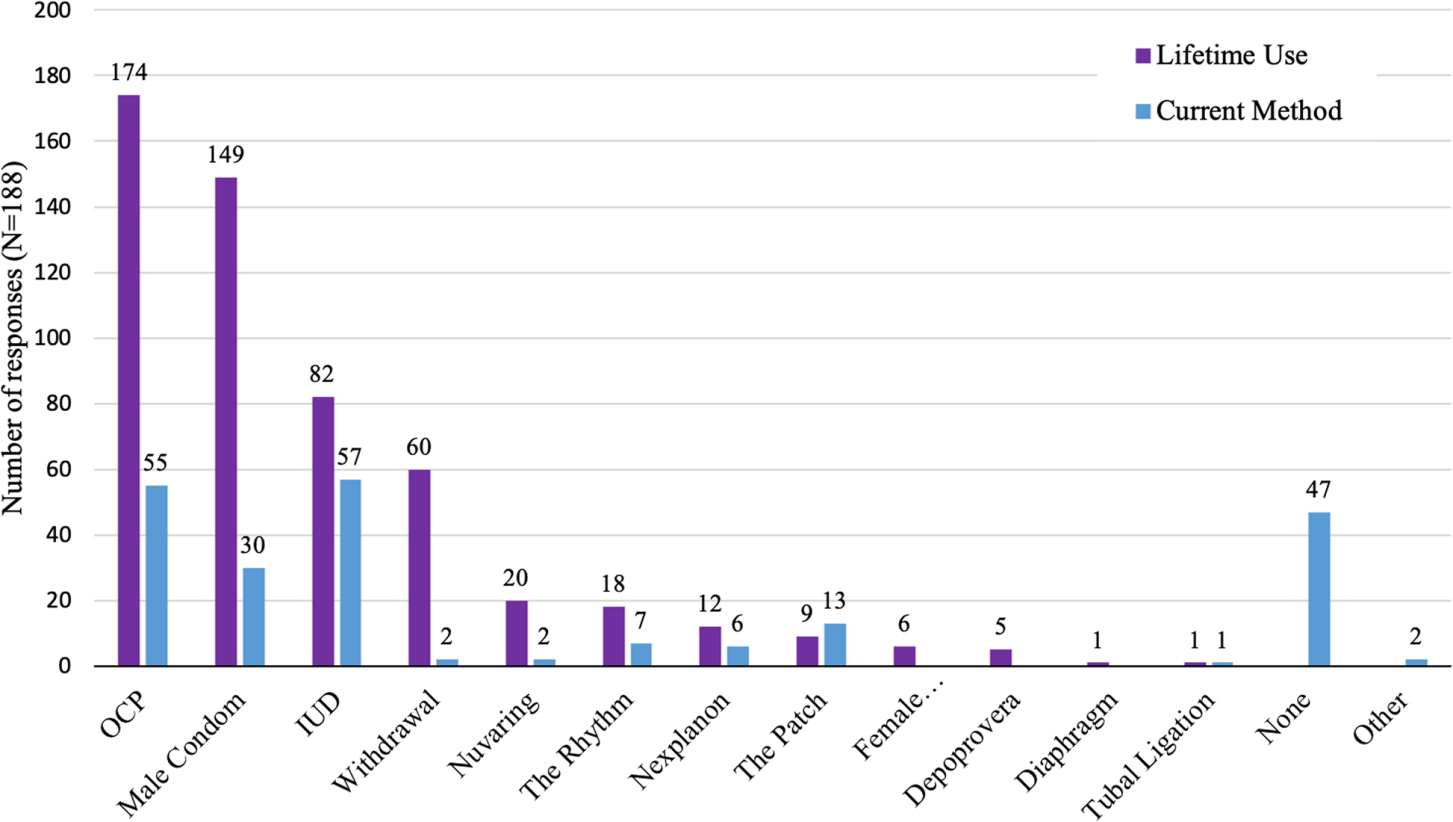
Contraception methods used by participants, (*N*=188)

Figure 1 shows the different methods of contraception participants have used throughout their lifetimes and the method(s) they are currently using. The majority of participants (92.6%, 174/188) have used OCPs at some point in their lifetime. At the time of completing the survey the most popular contraception method was IUD, with 30.3% (57/188) of participants currently using an IUD.

Table 3 shows the responses to the question “In your lifetime, have you experienced any of the following side effects associated with your HC?”. The most common side effect reported was mood changes, with 43.6% (82/188) of participants experiencing mood changes related to their HC at some point in their life. A majority (51.6%, 97/188) of participants reported experiencing mood changes and/or sexual side effects associated with HC at some point in their life. 

When asked which side effect was the most bothersome, 43 participants spontaneously mentioned psychological side effects including 2 participants who mentioned suicidal thoughts. Sixteen participants spontaneously mentioned sexual side effects including loss of libido and anorgasmia

**Table 3:** Lifetime incidence of side effects of HC in all participants (total) and comparing participants with a psychiatric history and those without psychiatric history. *P* values represent a comparison of means between the two groups (separated based on psychiatric history) using a t-test

**Side Effect**	**Total (*N*** ** = 188)**	**No Psychiatric History (*N*** ** = 98)**	**Psychiatric History (*N*** ** = 80)**	***P*** ** value**
	**n (%)**	**n (%)**	**n (%)**	
Mood Changes	82 (43.6)	29 (29.5)	49 (61.2)	0.000016*
Irregular Bleeding	72 (38.3)	41(41.8)	27 (33.8)	0.272
Weight Gain	64 (34.0)	27 (27.6)	34 (42.5)	0.229
Cramps/Pain	47 (25.0)	22 (22.4)	22 (27.5)	0.440
Loss of Libido/Sexual Satisfaction	42 (22.3)	21 (21.4)	19 (23.8)	0.714
Acne	38 (20.2)	18 (18.4)	18 (22.5)	0.497
No Side Effects	38 (20.2)	23 (23.4)	13 (16.3)	0.235
Headaches	10 (5.3)	3 (3.1)	7 (8.8)	0.102
Nausea	5 (2.7)	1 (1.0)	4 (5.0)	0.111
Heavy Bleeding	3 (1.6)	2 (2.5)	0	0.117
Clots	2 (1.1)	1 (1.0)	1 (1.3)	0.886
Other	28 (14.9)			

Table 4 displays quotes from participants about their experiences with HC and its side effects. Several themes in this qualitative data were identified. Fifty-five participants mentioned the burden of mood changes with HC. One participant discussed the mental toll of birth control: “When I was PMSing while on the hormonal [BC] pill, my anxiety became debilitating. … I have yet to find a long term contraceptive that works for me without taking a toll on my mental stability.” Sexual dissatisfaction and loss of libido were also mentioned by 22 participants. Additionally, 12 participants mentioned migraines and five mentioned lack of discussion about the increased risk of blood clots from HC.

**Table 4:** Key themes and quotes pertaining to participants’ personal experiences with contraception methods from qualitative data

**Theme**	**# of Responses**	**Selected Quotes**
Mood Changes	55	“Was I willing to risk my sanity to prevent pregnancy via birth control? (no)” “I experienced significant acne, spotting, and became very emotional, and it took my body about 6 months to adjust- all of which weren’t really discussed by my provider.” “[I had] mood changes and anxiety on implant. Nothing with oral contraceptive” “Mood symptoms were not affected by oral contraception but worsened with hormonal IUD so needed to discontinue.” * “Mood swings were terrible when I first went on the pill. It took several different types before I found one that worked best for me.” “Mood/psych symptoms and libido changes were never discussed when I started on hormonal OCPs at 18, and I did start developing mood and anxiety symptoms around the same time.” “I tried once this winter to go on [the pill], and within the first few days I was so depressed and I wouldn’t stop bleeding. I will never be on birth control again.” “When I was PMSing while on the hormonal birth control pill, my anxiety became debilitating. … I have yet to find a long term contraceptive that works for me without taking a toll on my mental stability.” “It has never felt very safe to try a new method of birth control, and it has not felt as if the provider would be able to adequately determine the actual cause of any symptoms or which methods might help alleviate them. I get a lot of “we can try it out, and see how it goes,“ which feels like gambling with my physical and mental health each time… I have often felt guilt about asking questions, especially when I was younger, because …providers have used a tone with me as though my worries, concerns, and questions are “not that big of a deal.“” “I experienced severe mood swings on the regular ‘pill’”

### Contraception counseling experiences

**Fig. 2:**
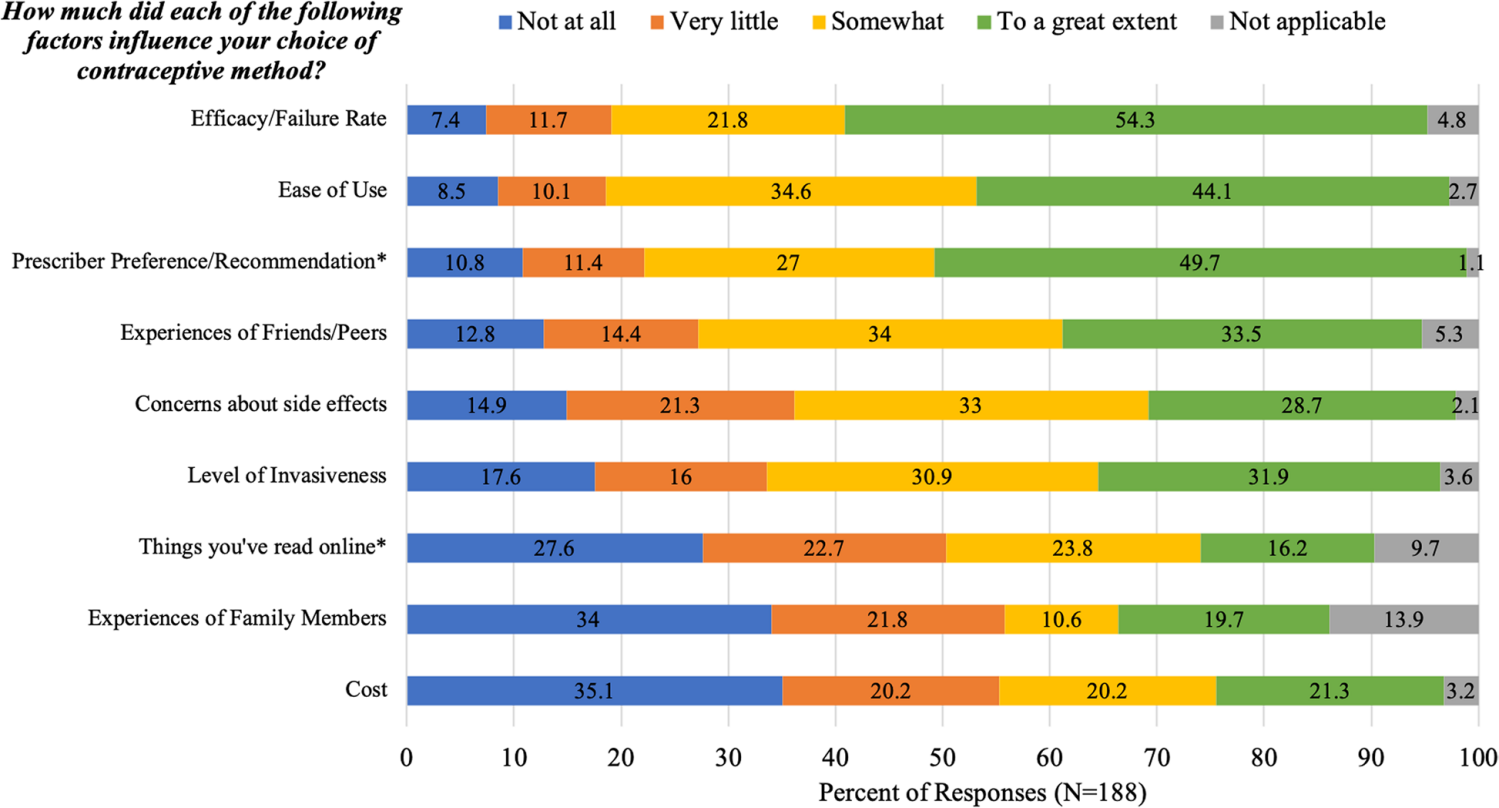
Participants’ responses to the question, “How much did each of the following factors influence your choice of contraceptive method?”, (*N* = 188)

Figure 2 shows how participants rated the importance of several factors in their choice of contraceptive method. *Efficacy/failure rate* was the most important factor with 54.3% (102/188) of respondents saying that efficacy influenced their decision “to a great extent.” Another important factor was *ease of use*, with 44.1% (83/188) of participants saying that ease of use influenced their decision “to a great extent.” *Prescriber preference/recommendation* also appeared to be an important factor with 49.7% (92/185) of respondents saying that prescriber preference influenced their decision “to a great extent.”

In the free text responses to the question about which factors influenced participants choice of contraceptive method, five participants noted “availability” as an important factor in their contraceptive decision. Additionally, 11 participants stated that they felt they did not have a choice in their contraceptive decision, noting that they just took what their provider prescribed without discussion of alternative methods.

**Fig. 3:**
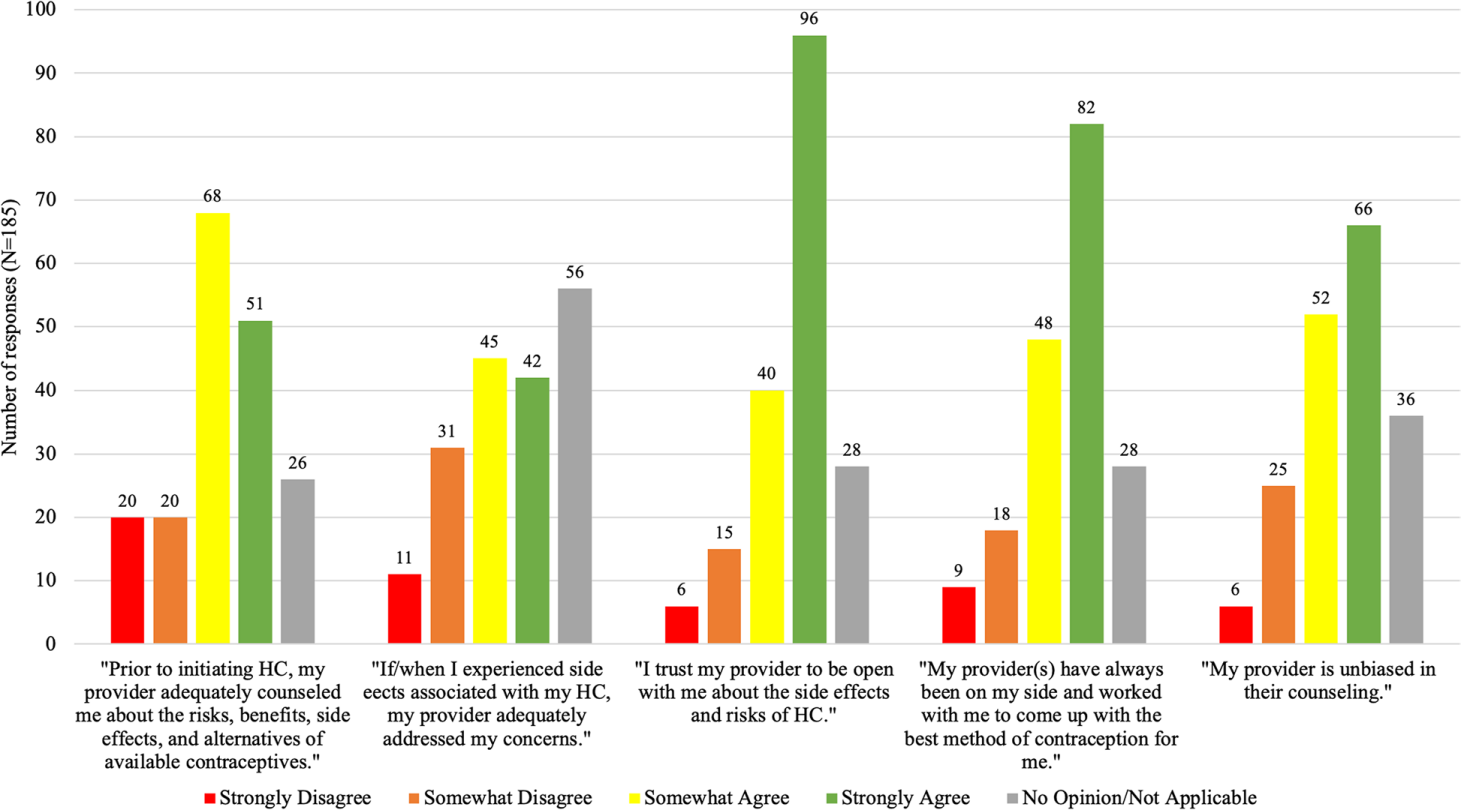
Participants’ responses to statements about their experiences with contraception counseling, (*N* = 185)

Figure 3 shows participants’ level of agreement (rated on a likert scale) with the several statements about their experiences with contraception counseling. For example, 40/185 (21.6%) of respondents disagree with the statement: “Prior to initiating HC, my provider adequately counseled me about the risks, benefits, side effects, and alternatives of available contraception.” Additionally, 42/185 (22.7%) disagreed with the statement: “If/when I experienced side effects associated with my HC, my provider adequately addressed my concerns.” The majority (151/182, 83%) of participants responded that their provider never mentioned the possibility of psychological or sexual side effects during contraception counseling. The majority (118/185, 64%) of responded agreed that their provider(s) are unbiased in their counseling and 74% (136/182) agreed that they trust their provider to be open with them about side effects and risks of HC.

Table 5 shows quotes about participants’ experiences with contraception counseling. We identified several important themes from this qualitative data. Eight participants mentioned not feeling heard by their provider. For example, one respondent stated, “I have a horrible gynecologist who infantilizes me and does not listen to my preferences for contraceptive methods or concerns” and another stated, “I experienced severe mood swings on the regular “pill” and when I mentioned this to my [gynecologist] he looked at me and said *‘sometimes that’s just life you have a bad day you have to learn to live with it’.”* Eighteen participants mentioned a lack of discussion about options during the contraceptive counseling experience. For example, one respondent stated “hormonal birth control sucked and beyond condoms or abstinence there was no other alternative offered.” Sixteen participants also mentioned the lack of discussion with their provider on the side effects of birth control. For example, one participant stated *“*I don’t feel as though I was adequately informed about the possible psychological impacts of birth control when I started.” Six individuals described a positive experience with birth control.

**Table 5:** Key themes and quotes pertaining to participant’s experiences with contraception counseling from qualitative data

**Themes**	**# of Responses**	**Selected Quotes**
Feeling dismissed by provider	8	“I experienced severe mood swings on the regular “pill” and when I mentioned this to my GYN he looked at me and said ‘sometimes that’s just life you have a bad day you have to learn to live with it’.” “Mood changes [were the worst side effect] and I was dismissed when I mentioned it to my GYN” “When I was PMSing while on the hormonal birth control pill, my anxiety became debilitating. I tried to talk to my doctor about switching off the pill and to an IUD, but did not feel heard and ended up off the pill and without an IUD.” “I’ve always taken BC pills… I recently tried to talk to my gyn about non hormonal options and she told me there weren’t really any good ones. Full stop. I’ve never felt like a doctor really wanted to help find the best unique solution for me.” “I have a horrible gynecologist who infantilizes me and does not listen to my preferences for contraceptive methods or concerns.” “I had a lot more questions than I felt comfortable asking. That may not be the case with all patients, but I wished the visit felt less routine and more focused on what, to me, was a big step in my life.” “Much of the counseling I have received has felt rushed, and like the provider is playing a guessing game along with me but they don’t have much time to collect detailed info. It has never felt very safe to try a new method of birth control, and it has not felt as if the provider would be able to adequately determine the actual cause of any symptoms or which methods might help alleviate them. I get a lot of “we can try it out, and see how it goes,“ which feels like gambling with my physical and mental health each time… I have often felt guilt about asking questions, especially when I was younger, because providers have used a tone with me as though my worries, concerns, and questions are “not that big of a deal.” “[I] was barred from getting my tubes tied, several times at different ages, even though I have never wanted children. I can only hope for better options in the future or a doctor that listens.”
Lack of Discussion of Side Effects	16	“Providers shouldn’t automatically refill hormonal contraceptive prescriptions without asking about complications/side effects/contraindications: shortness of breath, leg swelling, smoking status etc.” “I first started hormonal contraception when I was a teenager over 20 years ago. I received very little counseling at the time and side effects were almost completely dismissed.” “Looking back I don’t believe I was told the truth w/regard to side effects of oral contraceptives.” “I don’t feel as though I was adequately informed about the possible psychological impacts of birth control when I started.” “I didn’t know how to answer the counseling q’s because my original gyno did a terrible job of explaining anything and addressing the concerns of high bp after taking the pill. She just said you’re going to college so you should go on the pill.” “Mood/psych symptoms and libido changes were never discussed when I started on hormonal OCPs at 18, and I did start developing mood and anxiety symptoms around the same time.”
Lack of discussion on options	18	“my original gyno did a terrible job of explaining anything and addressing the concerns of high bp after taking the pill. She just said you’re going to college so you should go on the pill.” “I was not counseled on all contraceptive options when I was first given birth control, only pills.” “I’ve changed providers multiple times, by far, the worst in terms of education was the first gynecologists’ I saw. Perhaps fewer options were available, but I just accepted that the pill was the only option other than condoms. I don’t think I was adequately informed about the available options such as an IUD, or the even lower dose versions of the pill. Even as an adult, most of the information I have has been self discovered, and then discussed with my physician.” “Hormonal birth control sucked and beyond condoms or abstinence there was no other alternative offered.” “I’ve always taken [the] BC pill… I recently tried to talk to my gyn about non hormonal options and she told me there weren’t really any good ones. Full stop. I’ve never felt like a doctor really wanted to help find the best unique solution for me.” “I feel like providers don’t talk enough about non-hormonal options if mood is affected. I [currently] have a non-hormonal iud, which I really had to talk my doctor into agreeing with me, and my moods have seen an improvement since I’ve been using it.” “I’ve found the most support in my contraceptive experience from my friends, and I provide that support as well. I also work in the psych field and there is no course or even single lesson on contraception and its potential impact on mood/hormones that interplay with psych meds or mental health experiences aside from 5 minutes on the interaction of birth control methods and mood stabilizers. Big area to expand on here, I would love more education and awareness around this topic.” “I can only hope for better options in the future.”
Positive Experiences	6	“My experience with oral contraceptives has been positive for me.” “My experience with hormonal birth control pills has been extremely positive and I have no desire to change contraceptive methods.” “I have so far been happy with this choice.” “Doctors (primary care) and gynecologists have been wonderful at advising me about contraception.” “My experience was excellent.”

### Psychiatric history

Regarding past psychiatric history, 44.9% (80/178) of participants responded that they had suffered from and/or been treated for a psychiatric condition. The most common psychiatric condition was anxiety, with 69.6% (55/79) of respondents with a psychiatric history reporting a history of anxiety. Other psychiatric history is displayed in Table 6. The mean age at which participants were diagnosed with psychiatric conditions was 19.7 (SD: 5.4, Range: 4–33, *N* = 77).

**Table 6:** Psychiatric histories and responses to HC

	n	%
**History of Psychiatric Illness (** ***N*** ** = 178)**	80	44.9
**Specify: (** ***N*** ** = 79)**		
Anxiety	55	69.6
Depression	53	67.1
Post Traumatic Stress Disoder (PTSD)	6	7.6
Attention Deficit Hyperactivity Disorder (ADHD)	6	7.6
Obsessive Compulsive Disorder (OCD)	6	7.6
Eating Disorder	5	6.3
Bipolar Disorder	3	3.8
**Did you notice any change in your psychiatric symptoms with HC? (** ***N*** ** = 80)**		
Symptoms worsened with HC	31	38.8
No change in symptoms with HC	16	20.0
Symptoms improved with HC	9	11.2
Unsure/Not applicable	27	33.8

#### Psychiatric History and Mood Changes with HC

Of the participants who reported experiencing mood changes as a side effect of HC, 62.8% (49/78) reported a history of being treated for and/or diagnosed with a psychiatric illness. Of the 80 participants (Table 6) who reported a history of psychiatric illness, 61.3% (49/80) reported experiencing mood changes as a side effect of HC. Among the 98 participants who denied a history of psychiatric illness, 29.6% (29/98) reported experiencing mood changes as a side effect of HC. The rate of mood changes associated with HC was significantly higher amongst participants with a psychiatric history compared to those without a psychiatric history (*p* < 0.00002).

For those patients who reported a history of psychiatric illness, 38.8% (31/80) responded that their psychiatric symptoms worsened with HC while 11.2% (9/80) responded that their symptoms improved with HC. Of note, three participants responded that their mood symptoms both worsened *and* improved with HC.

## Discussion

This is one of the first studies to investigate patients’ subjective experiences with HC side effects and with contraception counseling. In this study, 43.6% of participants reported experiencing mood changes as a side effect of HC. While we cannot draw any conclusions about causality based on our data, it is certainly notable that in our sample, mood changes were among the most common perceived side effects of HC. Meanwhile, existing guidelines for providers on contraception counseling fail to mention the possibility of psychological side effects associated with HC and offer little guidance on how to counsel patients about the possibility of these types of side effects [18–20] or respond to patients who voice these concerns.

As such, it is unsurprising that the vast majority of participants in this study (83%) stated that their providers made no mention of the possibility of psychological or sexual side effects during contraception counseling. Anticipatory guidance has been shown to increase medication adherence and patients’ trust in their providers and the health care system as a whole [21]; however, our data suggests a disconnect between existing literature on the subject and patients’ experiences. While providers play an important role in patients’ contraceptive decisions, minimizing the potential for adverse mood changes with HC has the potential to erode patient’s trust in their providers. This concern is borne out in our data. Nearly 17% of participants were concerned that their providers were biased, 22.7% did not feel adequately supported by their provider if/when they experienced side effects with HC, and 11.4% did not trust their provider to be open with them about the side effects and risks of HC. While the majority of participants reported trusting their providers, it is concerning that 1 in 5 participants felt that they were not adequately supported by their providers when they experienced side effects and this merits further investigation.

We found that patients’ choice of contraception was heavily influenced by the experiences of their peers. One third (33.5%) of participants stated that experiences of friends/peers influenced their choice of contraceptive method to a great extent and an additional 34% stated their peers’ experience somewhat influenced their choice. This is in line with existing literature which points to the social network as an important source of information for women when choosing contraceptive methods [22]. As anecdotal reports of psychological side effects of HC circulate in the media, patients may have understandable concerns about the impact of HC on their mood. Providers need to be prepared to address these concerns in a balanced manner that validates patients’ concerns.

While the existing literature around mood-related side effects of HC is limited and largely inconclusive, several reviews have suggested that patients with a history of affective disorders are at increased risk of psychological side effects with HC [13, 16]. We found that 63% of participants who experienced mood changes as a side effect of HC had a history of psychiatric illness. Reported rates of mood changes associated with HC were significantly higher among participants with a history of psychiatric illness (61.3%) compared with participants with no history of psychiatric illness (29.6%). Moreover, in people with a history of psychiatric illness, 39% reported that their symptoms worsened with HC while only 11% that their symptoms improved with HC. These findings support the hypothesis that people with underlying mood disorders may be at increased risk of psychological side effects with HC. It is worth noting, though, that 37% of participants who experienced mood changes with HC reported no history of psychiatric illness, suggesting that psychological side effects with HC may not be limited to patients with psychiatric illnesses.

Interestingly, 88% of participants used OCPs as their first form of HC, however, at the time of survey completion, only 29% of participants were currently using an OCP and 30% (59/188) were currently using an IUD. A prospective study of 79 women using combined OCPs found that emotional and sexual side effects were the best predictors of discontinuation or switching HC methods [2]. Sanders et al. found that 33% of participants spontaneously cited emotional side effects as a reason for discontinuing or switching methods. In our study, the most commonly cited reason for discontinuing or switching HC methods was side effects (48.3%). While we did not specifically ask patients which side effects led them to switch contraceptive methods, we did ask participants which side effects were most bothersome. In response to this question, 43 participants spontaneously mentioned psychological side effects, including two participants who mentioned suicidal thoughts with HC. Sixteen participants spontaneously mentioned sexual side effects including loss of libido and anorgasmia. These findings suggest that psychological side effects, at least in part, may have impacted HC users’ decisions to switch from OCPs to an alternative method of contraception. Some literature suggests that OCPs carry higher-rates of hormonal side effects including mood changes and libido, compared with IUDs, given that OCPs involve higher levels of systemic hormones while IUDs offer non-hormonal and localized lower-dose hormone options [13].

### Limitations

This study has several limitations. A relative lack of diversity in our sample with regards to race/ethnicity, education level, and age, may limit the generalizability of our findings. Conversely, the wide range of ages in this sample means that some participants may have been taking HC over 20 years ago, when standard doses of OCPs were much higher and the side effect burden may have been greater. There may also be a response bias in our sample. For example, it is possible that people who have had negative experiences with HC were more likely to respond to the survey. Additionally, a relatively high percentage of respondents reported a history of psychiatric illness compared to the general population. While this could represent response bias, it is worth noting that the most common psychiatric illnesses reported were anxiety (70%) and depression (67%). Psychiatric history was not obtained using structured screening or diagnostic tools. Many participants with self-reported anxiety and depression may not meet DSM-V criteria for Generalized Anxiety Disorder or Major Depressive Disorder. It should also be noted that this survey was administered in late 2021 and early 2022, during the COVID-19 pandemic. It makes sense that participants would endorse higher rates of anxiety and depression in the wake of an international public health crisis.

Other limitations include issues with the survey instrument itself. For example, despite our efforts to limit the length of the survey, several participants failed to complete the entire survey. Some participants had questions about language used in the survey including language regarding their sexual and/or gender identities, and the definition of contraception counseling. Another major limitation of the survey instrument is that we did not ask participants to differentiate between hormonal and non-hormonal IUDs. We also lack specific information about the types of HC participants were prescribed, the doses, and which HC methods were associated with particular side effects. Lastly, we did not specifically ask about *which* side effects caused people to switch/discontinue methods.

## Conclusion

There exists considerable debate about the relationship between HC and mood-related side effects. Much of the literature on this subject is conflicting and inconclusive. Our findings suggest that mood changes are among the most common perceived side effects of HC and may be among the leading reasons for discontinuing or switching contraception methods in our sample. Furthermore, this data suggests that many patients feel like they were not adequately counseled about the possibility of psychological side effects. Guidelines for providers about how to counsel patients about the risks of psychological and sexual side effects of HC are sorely lacking and tend to err on the side of minimizing patients’ concerns about these types of side effects. For example, a table in UpToDate showing examples of contraception counseling exchanges suggests that a provider could respond to a patient with concerns that “the NuvaRing made her friend ‘feel crazy’” by saying: “That’s too bad your friend had that experience. I haven’t heard of that before, and I can tell you it definitely doesn’t happen frequently. In general, no contraceptive methods have an effect on mental health. Since everyone is different, I wouldn’t expect the same thing that happened to your friend to happen to you” [18]. This exchange demonstrates and promotes the minimization and dismissal of patients’ concerns about psychological side effects with HC and fails to incorporate the available data.

This disconnect between patients’ experiences and provider counseling may erode patients’ trust in their providers and the healthcare system as whole. Existing guidelines on the subject of contraception counseling fall short when it comes to discussion of psychological side effects with HC. We suggest that providers should consider psychiatric history and mood symptoms when prescribing contraception, discuss the possibility of mood changes with their patients, and assess for mood symptoms during follow-up appointments, especially in the most vulnerable patients. If providers are not equipped to discuss psychological side effects of HC with their patients by validating and addressing their concerns with shared-decision making, patients may be more likely to turn to non-hormonal or less effective methods of family planning including fertility awareness (also known as “The Rhythm Method”) and withdrawal. More research including prospective, carefully controlled studies are required to better understand the relationship between HC and mood symptoms. Additionally, our findings suggest a need for larger scale studies on the topic of patients’ experiences with contraceptive counseling. In the meantime, providers should be aware of their patients’ concerns and prepared to respond to them with validation and care.

## Supplementary Information

**Additional file 1.** Survey Instrument.

## Data Availability

The datasets used and/or analyzed during the current study are available from the corresponding author on reasonable request.

## Abbreviations

ADHD: attention deficit hyperactivity disorder
BC: birth control
HC: hormonal contraception
IRB: institutional review board
IUD: intrauterine device
OCP: oral contraceptive pill
OCD: obsessive compulsive disorder
PTSD: post traumatic stress disorder
PMS: premenstrual syndrome
SD: standard deviation
