# Supplementary material for: Psychological side effects of hormonal contraception: a disconnect between patients and providers
Source: Contracept Reprod Med. 2023 Jan 17;8:9. doi: 10.1186/s40834-022-00204-w (PMC9842494; doi:10.1186/s40834-022-00204-w)
Supplement: Supplementary file 1 — Additional file 1. Survey Instrument. [file 40834_2022_204_MOESM1_ESM.pdf]

Patients Experiences with Hormonal Contraception and Contraception Counseling

Demographics

What is your gender identity?

- ◯ Cisgender female
- ◯ Cisgender male
- ◯ Transgender female
- ◯ Transgender male
- ◯ Non-binary
- ◯ Other
- ◯ Prefer not to say

Other

____

How old are you?

____

Which category best describes you? (select all that apply)

- ☐ White
- ☐ Black or African American
- ☐ Asian
- ☐ Native-American
- ☐ Middle Eastern or North African
- ☐ Native Hawaiian or Pacific Islander
- ☐ Hispanic/Latino/Spanish
- ☐ Other
- ☐ Prefer not to say

Other

____

What is your current occupation?

- ☐ Undergraduate student
- ☐ Graduate Student
- ☐ Service industry (Food service, retail, etc.)
- ☐ Healthcare worker (Nurse, Doctor, etc.)
- ☐ Businessperson (Law, Sales, Finance, etc.)
- ☐ Entertainment/Arts
- ☐ Homemaker
- ☐ Unemployed
- ☐ Other (please specify)

Other

____

What is the highest level of education you have completed?

- ◯ High School
- ◯ Undergraduate Degree (Bachelor's or Associates)
- ◯ Masters degree
- ◯ Doctorate Degree
- ◯ Other

Other

____

What is your sexual orientation?	◯ Heterosexual<br>◯ Homosexual<br>◯ Bisexual or Pansexual<br>◯ Asexual<br>◯ Queer<br>◯ Other
Other	______________________
Have you ever been pregnant?	◯ Yes<br>◯ No
How many times have you been pregnant?	______________________
Have you ever had an elective abortion?	◯ Yes<br>◯ No
How many times have you given birth?	______________________
What is your current relationship status?	☐ Married<br>☐ Single, sexually active<br>☐ Single, not sexually active<br>☐ Divorced<br>☐ Other
Other	______________________
Have you ever used hormonal contraception (eg. oral contraceptive pills, implant, the patch, injections, IUD)?	◯ Yes<br>◯ No
Have you ever received contraception counseling from a healthcare provider?	◯ Yes<br>◯ No<br>(Contraception counseling occurs when a healthcare provider educates patients and answers questions about various methods of birth control and the risks/benefits associated with different methods.)
Thank you for taking this survey! You may skip to the end of the survey and click "submit".	
At what age did you start using contraception?	______________________
What was the first kind of hormonal contraception you used?	☐ Oral Contraceptive Pills<br>☐ IUD (Intrauterine device)<br>☐ Depo-provera (The Shot)<br>☐ Nexplenon (Implant)<br>☐ The Patch<br>☐ NuvaRing<br>☐ Other<br>(For this question, contraception does not include the use of barrier methods such as condoms.)

Other	__________________________________
Who prescribed you your first hormonal contraception?	◯ Gynecologist<br>◯ Primary Care Doctor<br>◯ Pediatrician<br>◯ Other
Other	__________________________________
For what reason(s) did you start using hormonal contraception? (Select all that apply)	☐ Pregnancy prevention<br>☐ Acne<br>☐ Heavy periods<br>☐ Irregular periods<br>☐ Painful periods<br>☐ Premenstrual symptoms<br>☐ Other
Other	__________________________________

How much did each of the following factors influence your choice of contraceptive method?

	Not at all	Very little	Somewhat	To a great extent	Not applicable
Prescriber Preference/Recommendation	◯	◯	◯	◯	◯
Experiences of Friends/Peers	◯	◯	◯	◯	◯
Experiences of Family Members	◯	◯	◯	◯	◯
Concerns about side effects	◯	◯	◯	◯	◯
Cost	◯	◯	◯	◯	◯
Ease of Use	◯	◯	◯	◯	◯
Efficacy/Failure Rate	◯	◯	◯	◯	◯
Things you've read online	◯	◯	◯	◯	◯
Level of invasiveness	◯	◯	◯	◯	◯

What other factors influenced your contraception decisions? ______________________________

What types of contraception have you used in your lifetime? (select all that apply)

- ☐ Intrauterine device (IUD)
- ☐ Oral Contraceptive Pills
- ☐ Male Condoms
- ☐ Female Condoms
- ☐ Nuvaring
- ☐ Nexplenon (Implant)
- ☐ Depo-Provera (injection)
- ☐ The Patch
- ☐ Withdrawal
- ☐ Fertility awareness method (aka The Rhythm)
- ☐ Other

Other ______________________________

What type of contraception are you currently using?

- ☐ Intrauterine device (IUD)
- ☐ Oral Contraceptive Pills
- ☐ Male Condoms
- ☐ Female Condoms
- ☐ Nuvaring
- ☐ Nexplenon (Implant)
- ☐ Depo-Provera (Injection)
- ☐ Withdrawal
- ☐ The Patch
- ☐ Fertility awareness method (aka The Rhythm)
- ☐ Other
- ☐ Not currently using contraception

Other ______________________________

If you have switched contraception methods or stopped using hormonal contraception, what was your reason?

- ☐ Desire to become pregnant
- ☐ No longer sexually active
- ☐ Loss of access
- ☐ Difficulty remembering a daily pill
- ☐ Side Effects
- ☐ Other

Other

____

In your lifetime, have you ever experienced side effects associated with your contraception? (select all that apply)

- ☐ Acne
- ☐ Cramps/Pain
- ☐ Irregular menstrual bleeding
- ☐ Loss of libido/sexual satisfaction
- ☐ Mood changes
- ☐ Weight gain
- ☐ Other (please specify)
- ☐ No side effects

Other

____

What side effect(s) bothered you the most?

____

The following questions will assess your experience(s) with contraception counseling prior to initiating contraception and throughout your lifetime. Please rate how much you agree with the following statements.

	Strongly disagree	Somewhat disagree	Somewhat agree	Strongly agree	Not applicable/no opinion
"Prior to initiating hormonal contraception, my provider adequately counseled me about risks, benefits, side effects and alternatives of available contraceptives."	○	○	○	○	○
"If/when I experienced side effects associated with my hormonal contraception, my provider adequately addressed my concerns."	○	○	○	○	○
"I trust my provider to be open with me about the side effects and risks of hormonal contraception"	○	○	○	○	○
"My provider(s) have always been on my side and worked with me to come up with the best method of contraception for me."	○	○	○	○	○
"My provider is unbiased in their counseling"	○	○	○	○	○

During contraception counseling, did your provider ever discuss the possibility of psychological or sexual side effects?

○ Yes
○ No

What did they tell you?

____

	Very negative	Somewhat negative	Neutral, no opinion	Somewhat positive	Very positive	Not applicable, unsure
Overall, when you think about your experience(s) with hormonal contraception, how do you feel?	○	○	○	○	○	○

The following questions are about your mental health history. Please answer only if you feel comfortable.

Have you suffered from or been treated for a psychiatric condition?	◯ Yes<br>◯ No
Please specify	__________________________________
At what age were you first diagnosed with and/or treated for a psychiatric condition?	__________________________________
Did you notice any changes in your psychiatric symptoms with hormonal contraception?	☐ Yes, mood symptoms worsened with hormonal contraception<br>☐ Yes, mood symptoms improved with hormonal contraception<br>☐ No change in mood symptoms with hormonal contraception<br>☐ Unsure or not applicable
Is there anything else you would like to share about your experience with contraception and/or contraception counseling?	____
